# Usefulness of the N-Terminal of the Prohormone Brain Natriuretic Peptide in Predicting Acute Kidney Injury Requiring Renal Replacement Therapy in Patients Undergoing Heart Valve Surgery

**DOI:** 10.3390/medicina59122083

**Published:** 2023-11-27

**Authors:** Piotr Duchnowski, Witold Śmigielski

**Affiliations:** 1 Ambulatory Care Unit, Cardinal Wyszynski National Institute of Cardiology, Alpejska 42, 04-628 Warsaw, Poland; 2 Cardinal Wyszynski National Institute of Cardiology, 04-628 Warsaw, Poland

**Keywords:** acute kidney injury (AKI), heart valve surgery, postoperative complications, N-terminal of the prohormone brain natriuretic peptide (NT-proBNP), renal replacement therapy

## Abstract

*Background and Objectives*: By definition, acute kidney injury (AKI) is a clinical syndrome diagnosed when the increase in serum creatinine concentration is >0.3 mg/dL in 48 h or >1.5-fold in the last seven days or when diuresis < 0.5 mL/kg/h for a consecutive 6 h. AKI is one of the severe complications that may occur in the early postoperative period in patients undergoing heart valve surgery, significantly increasing the risk of death. Early implementation of renal replacement therapy increases the chances of improving treatment results in patients with postoperative AKI. The study assessed the predictive ability of selected preoperative and perioperative parameters for the occurrence of postoperative AKI requiring renal replacement therapy in the early postoperative period in a group of patients with severe valvular heart disease. *Materials and Methods*: A prospective study was conducted on a group of patients undergoing consecutive heart valve surgeries. The primary endpoint was postoperative AKI requiring renal replacement therapy. AKI was diagnosed with an increase in serum creatinine > 0.3 mg/dL in 48 h or >1.5-fold in the previous 7 days and/or a decrease in diuresis < 0.5 mL/kg/h for 6 h. The observation period was until the patient was discharged home or death occurred. Logistic regression analysis was used to assess which variables were predictive of primary endpoint, and odds ratios (OR) were calculated with a 95% confidence interval (CI). Multivariate analysis was based on the result of single factor logistic regression, i.e., to further steps, all statistically significant variables were taken into consideration. *Results:* A total of 607 patients were included in the study. The primary endpoint occurred in 50 patients. At multivariate analysis: NT-proBNP (OR 1.406; 95% CI 1.015–1.949; *p* = 0.04), CRP (OR 1.523; 95% CI 1.171–1.980; *p* = 0.001), EuroSCORE II (OR 1.090; 95% CI 1.014–1.172; *p* = 0.01), age (OR 1.037; 95% CI 1.001–1.075; *p* = 0.04) and if they stayed in the intensive care unit longer than 2 days (OR 9.077; 95% CI 2.026–40.663; *p* = 0.004) remained the independent predictors of the primary endpoint. The mean preoperative NT-proBNP level was 2063 pg/mL (±1751). Thirty-eight patients with AKI requiring renal replacement therapy died in intrahospital follow-up. *Conclusions:* The results of the presented study indicate that a high preoperative level of NT-proBNP and postoperative hemodynamic instability may be associated with a significant risk of a postoperative AKI requiring renal replacement therapy. The results of the study may also suggest that qualifying for heart valve surgery earlier may be associated with improved prognosis in this group of patients.

## 1. Introduction

According to the definition, acute kidney injury (AKI) is a clinical syndrome diagnosed when the increase in serum creatinine concentration is >0.3 mg/dL in 48 h or >1.5-fold in the last seven days or when diuresis < 0.5 mL/kg/h for consecutive six hours. The causes of AKI can be divided into three groups: prerenal, parenchymal and postrenal. Prerenal AKI is caused by impaired renal perfusion, most often as a result of decreased renal perfusion due to a decrease in circulating blood volume, cardiac output, blood pressure, or renal artery disease, resulting in a decrease in blood flow to the glomerulus and, as a result, a decrease in glomerular filtration rate. The main factors leading to prerenal kidney damage include, among others, low cardiac output in the course of heart muscle diseases such as acute myocardial infarction, heart failure, cardiac arrhythmias or valvular heart disease, and pulmonary embolism, mechanical ventilation of the lungs with positive pressure, or renal artery occlusion, fluid loss through the gastrointestinal tract and kidneys or hemorrhage [1,2,3,4,5]. The initial treatment for AKI includes the removal of the cause and hemodynamic compensation of the patient. According to KIDGO recommendations from 2012, the decision to start renal replacement therapy in patients with AKI should be made in the event of conditions that can be modified as a result of this treatment and based on the direction of changes in laboratory tests, and not only based on rigidly established threshold values of serum creatinine concentration [6,7,8]. 

AKI is a serious complication that may occur in the early postoperative period in patients undergoing heart valve surgery, significantly increasing the risk of death. The main aim of renal replacement therapy in patients with AKI in the early postoperative period is to replace renal function during renal failure and to facilitate spontaneous regeneration of the renal parenchyma. Knowledge of the predictors of postoperative AKI requiring renal replacement therapy in patients undergoing heart valve surgery is extremely important, as it enables the identification of patients particularly at risk of this serious complication in the preoperative period, optimal preparation of high-risk patients for cardiac surgery, special supervision of high-risk patients in the perioperative period, and in the case of occurrence of AKI in the postoperative period, making a quick decision to implement renal replacement therapy, which increases the chance of improving treatment results. However, in the available literature, knowledge about the factors predicting postoperative AKI that is refractory to conservative treatment and thus requiring renal cell therapy is limited. So far, predictors of perioperative AKI in patients undergoing cardiac surgery include preoperative high creatinine levels, end-stage renal disease, 2-2 haptoglobin phenotype, advanced age, presence of comorbidities such as diabetes and congestive heart failure, generalized atherosclerosis, nitric oxide, cyanotic heart disease, long duration of cardiac surgery, increased preoperative RDW level or increased postoperative lactate level [5,9,10,11,12].

The validation of natriuretic peptides (NP)—such as N-terminal proBNP (Nt-proBNP)—as a biomarker of heart failure (HF) has changed everyday clinical practice in the last decades. Currently, the Nt-proBNP molecule has become so important in the diagnosis of HF that it is now included in the definition of the disease. Nt-proBNP also emerged as a powerful prognostic biomarker, identifying patients at the highest risk of events. The increase in NT-proBNP concentration in blood serum precedes the severity of heart failure symptoms and hospitalization. In turn, reducing the level of Nt-proBNP in blood serum results in clinical improvement [13]. 

So far, the available literature lacks clear information on the predictive ability of the preoperative Nt-proBNP level in predicting the occurrence of postoperative AKI requiring renal replacement therapy in patients undergoing heart valve surgery. It has been shown that an increased level of Nt-proBNP is an independent predictor of the occurrence of postoperative AKI in the general group of adult patients treated with cardiac surgery as well as in the pediatric group; otherwise, it has been demonstrated that elevated Nt-proBNP values are associated with a prolonged stay in an intensive care unit after cardiac surgery for congenital heart defects [14,15,16].

Due to the significant clinical problem of postoperative AKI, its quite frequent occurrence and its unfavorable prognosis, the main aim of the study was to evaluate selected preoperative and perioperative parameters, including the preoperative NTpro-BNP level, in terms of predicting the occurrence of AKI requiring renal replacement therapy in the early postoperative period.

## 2. Methods

A prospective study was conducted on a group of consecutive patients undergoing qualified and undergoing cardiac surgery due to valvular heart disease (aortic stenosis, aortic regurgitation, mitral valve stenosis, mitral regurgitation and tricuspid valve disease) with or without concomitant coronary artery disease at the Cardinal Wyszynski National Institute of Cardiology in Warsaw, Poland. The exclusion criteria were lack of consent of the patient to participate in the study, age below 18, preoperative end-stage renal failure requiring renal replacement therapy, active neoplastic disease, significant stenosis in the cerebral arteries and the porcelain aorta. The day before the cardiac surgery treatment, blood was taken from each patient to determine the level of biomarkers. Blood count parameters were analyzed using the Sysmex K-4500 automatic analyzer ((Sysmex, Kobe, Japan). NT-proBNP levels were determined using the electrochemiluminescent immunoassays Elecsys 2010 (Roche, Munich, Germany). Cardiac surgery was performed by median sternotomy under general anesthesia. Induction of anesthesia was performed using intravenous etomidate, pancuronium and fentanyl. Maintenance of general anesthesia was performed using intravenous propofol, fentanyl and inhalation sevoflurane. Each procedure was performed with the use of a cardiopulmonary bypass and aortic transverse clamp applied. The perfusion rate during cardiopulmonary bypass was 2.4–2.5 L/min/m^2^. In turn, the volume of the primary filling was obtained using crystal priming of 1200–1500 mL, which is a solution of colloid, crystalloids, mannitol and heparin. 

Moreover, patients received an initial dose of 15–20 mL/kg for cold-blooded cardioplegia during surgery, followed by booster doses of 5–10 mL/kg every 20 min. The primary endpoint was AKI observed in the early perioperative period, requiring renal replacement therapy in the form of continuous veno-venous hemodiafiltration. Acute kidney injury was diagnosed with an increase in serum creatinine > 0.3 mg/dL in 48 h or >1.5-fold in the previous 7 days and/or a decrease in diuresis < 0.5 mL/kg/h for 6 h. Creatinine level and 24 h urine collection were assessed daily in each patient in the first days after cardiac surgery. The decision to use renal replacement therapy was made by the team of anaesthesiologists caring for the patient with AKI in the early postoperative period based on changes in laboratory test results and clinical conditions. The observation period was until the patient was discharged home or death occurred (the early observation period refers to the postoperative stay in the intensive care unit and then in the cardiac surgery department until discharge from the hospital). The study protocol was approved by the Bioethics Committee operating at the National Institute of Cardiology in Warsaw, Poland—study number 2.32/VI/18. Each patient recruited for the study gave written consent before enrollment. 

### Statistical Analysis

The collected data are presented as median +/− quarter deviation and frequency (%). Intergroup cooperation was conducted using the Mann–Whitney U test in the cases of quantitative variables and the chi-square test in the cases of qualitative variables. Pre- and intra- and postoperative variables (listed in Table 1) were examined for an association with the primary endpoint. Logistic regression analyses were used to assess which variables were predictive of the primary endpoint, and the odds ratios (OR) were calculated with a 95% confidence interval (CI). Multivariate analysis was based on the result of single-factor logistic regression, i.e., to further steps, all statistically significant variables were considered. To improve the interpretation of the odds ratio, all quantitative variables were recoded to binary variables based on cut-off points established by using the ROC curves. Statistical data analyses were performed using STATISTICA software (version 13.0). Statistical significance was defined as *p* < 0.05 for all the analyses.

## 3. Results

**Figure 1:**
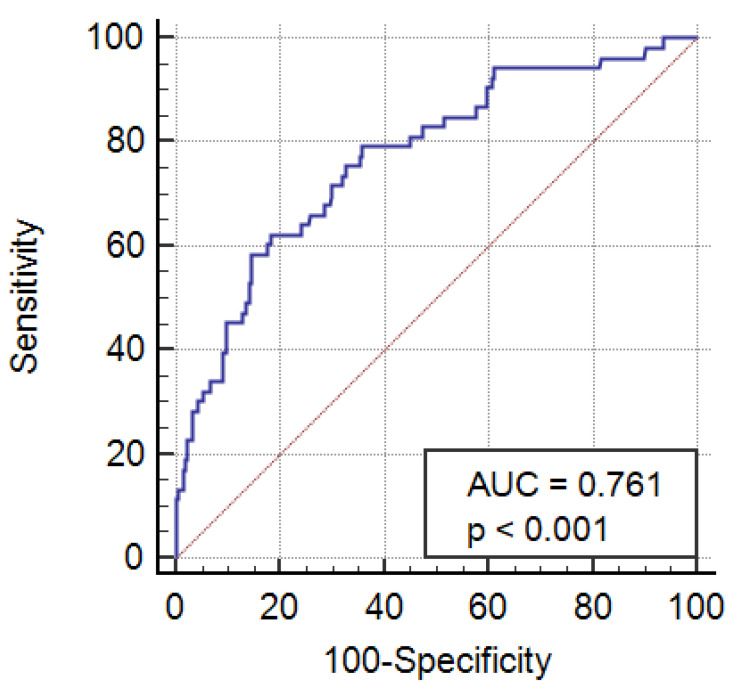
Area under receiver operating characteristic (ROC) curve of NT-proBNP for a renal replacement therapy following valve replacement/repair surgery.

The study included 607 patients undergoing valve/valves repair or replacement surgery. The characteristics of the patients are presented in Table 1. The average age was 63 years (±13). The mean preoperative NT-proBNP level was 2063 pg/mL (standard deviation [SD] ± 1751). One hundred eighty-six (30%) patients had preoperative chronic kidney disease (GFR < 60 mL/min/1.73 m^2^), but none of the patients included in the study required dialysis in the preoperative period. In the early postoperative period, 5- patients required renal replacement therapy (25 patients with preoperative chronic kidney disease), of which 45 patients had postoperative instability requiring prolonged administration of pressor amines, while 11 required mechanical circulatory support in the form of extracorporeal membrane oxygenation (ECMO) or intraaortic balloon pump (IABP) due to the development of post-cardiotomy shock. The average duration of hemodiafiltration was 4 days (±2). The statistically significant predictors of postoperative renal replacement therapy at univariate and multivariate analysis are presented in Table 2. At multivariate analysis: NT-proBNP (OR 1.406; 95% CI 1.015–1.949; *p* = 0.04), CRP (OR 1.523; 95% CI 1.171–1.980; *p* = 0.001), EuroSCORE II (OR 1.090; 95% CI 1.014–1.172; *p* = 0.01), age (OR 1.037; 95% CI 1.001–1.075; *p* = 0.04) and stayed in the intensive care unit longer than 2 days (OR 9.077; 95% CI 2.026–40.663; *p* = 0.004) and remained independent predictors of the primary endpoint. The optimal cut-off point for postoperative renal replacement therapy was calculated at >2464 pg/mL NT-proBNP (sensitivity 61%, specificity 81%). The area under the receiver operator characteristic curve for perioperative AKI for NT-proBNP is 0.761 (95% CI 0.25–0.795) (Figure 1). Thirty-eight patients with AKI requiring renal replacement therapy died in the intrahospital follow-up. The main cause of death was the increasing postoperative multiple organ dysfunction syndrome (MODS). The analysis showed a statistically significant correlation between the preoperative level of NT-proBNP and GFR value (r = −0.26, *p* < 0.001).

## 4. Discussion

Incidence of an acute kidney injury (AKI) in patients undergoing heart valve surgery is a fairly common complication observed in the early postoperative period, occurring with a frequency of about 3 to 34% [5,15,16]. Despite constant progress in intensive care, nephrology and dialysis, mortality in acute kidney injury remains high—in the presented study, out of 50 patients with postoperative AKI requiring renal replacement therapy, in-hospital death occurred in 38 patients. In addition, AKI prolongs hospital stay, increases the risk of developing chronic kidney disease, and is associated with increased post-hospital mortality. In order to improve the prognosis in patients with AKI, it is postulated to start renal replacement therapy before the occurrence of metabolic complications, such as hyperhydration unresponsive to treatment with diuretics, treatment-resistant hyperkalemia (serum potassium concentration > 6.5 mmol/L or lower in the presence of ECG changes); metabolic acidosis (pH < 7.1) or clinical signs of uremia (such as uremic pericarditis, uremic encephalopathy, uremic bleeding diathesis) [17]. Hemodiafiltration seems to be the gold standard for renal function replacement in AKI patients in the early postoperative period [18,19,20]. The use of knowledge about the predictors of postoperative AKI requiring the use of renal replacement therapy enables preoperative identification of patients at risk of postoperative kidney damage, special supervision of the patient both during surgery and in the early postoperative period, and early intensification of conservative treatment as well as the implementation of renal replacement therapy, which in turn may increase the chances of for the patient’s survival. 

Heart failure is a common disease entity observed in patients with postoperative AKI requiring renal replacement therapy in the early period after heart valve surgery [21,22,23]. In the above study, out of 50 patients who underwent continuous renal replacement therapy (CRRT) in the form of hemodiafiltration, 45 patients had persistent postoperative hemodynamic instability requiring prolonged administration of catecholamines, and 11 of the patients mentioned above required additional mechanical support due to the development of post-cardiotomy shock in the form of ECMO or IABP. The performed univariate logistic regression analysis confirmed postoperative hemodynamic instability as a predictor of postoperative AKI requiring renal replacement therapy. In the multivariate logistic regression analysis, independent predictors of AKI requiring renal replacement therapy were increased preoperative NT-proBNP concentration, age, CRP, EuroSCORE II, and staying in the intensive care unit longer than 2 days. Therefore, the results of the statistical analyses above indicating the predictive ability of the NT-proBNP parameter may be very valuable from the first contact with a patient with valvular heart disease. The incidence of valvular heart disease is constantly increasing, such as aortic stenosis or mitral valve regurgitation, among others, due to the aging of the human population. 

Choosing the optimal time for intervention, whether surgical or percutaneous, requires constant monitoring of patients [24]. The presence of symptoms and/or signs of left ventricular dysfunction in imaging studies are key guidelines for qualifying patients for invasive treatment of valvular heart disease. However, identifying symptoms can be particularly difficult, especially in older patients [25,26,27]. Therefore, readily available prognostic tools are sought for routine follow-up of patients with valvular heart disease. In recent decades, brain natriuretic peptide (BNP) and the N-terminal part of this prohormone (NT-proBNP) have become powerful biochemical markers reflecting cardiac function. BNP is synthesized by cardiomyocytes as a 108 amino acid prohormone termed proBNP. During the process of secretion of proBNP by cardiomyocytes, it is cleaved into the biologically active part of BNP consisting of amino acids from positions 77 to 108 and the remaining part of NT-proBNP consisting of amino acids 1 to 76 [28]. Currently, NT-proBNP is a recognized and widely used biomarker in diagnosing and assessing the progression of heart failure [29]. So far, elevated levels of natriuretic peptides such as BNP and the N-terminal fragment of its prohormone (NT-proBNP) have been shown to predict mortality and hospitalization in patients with severe aortic stenosis or mitral regurgitation. It was also shown that the severity of valvular heart disease and the New York Heart Association (NYHA) functional class are significantly correlated with higher absolute concentrations of NT-proBNP. A similar strength of correlation was also found between the level of NT-proBNP and the indexed size of the left atrium and the LV ejection fraction in all types of valvular heart disease. The available literature has also shown that serial determination of NT-proBNP is useful in patients with asymptomatic aortic stenosis as well as mitral regurgitation to optimize the timing of valvular intervention, which may be especially important if echocardiography is difficult to access. In addition, a significant correlation was found between the level of natriuretic peptides and the development of myocardial fibrosis in patients with valvular heart disease, most pronounced in patients with aortic stenosis, which was associated with worse outcomes also after valvular interventions [30,31,32,33,34].

The results presented study indicate that patients with high preoperative NT-proBNP values undergoing heart valve surgery may be at risk of postoperative AKI requiring renal replacement therapy. In a patient with severe symptomatic valvular disease, the left ventricular myocardium is overloaded by persistently high blood pressure and/or increased blood volume, forcing increased secretion of NT-proBNP by cardiomyocytes. Persistent long-term overload of the left ventricular muscle promotes the progressive degenerative process, as well as the development of heart failure [35,36,37]. The significant correlation between the preoperative levels of NT-proBNP and GFR may indicate that the increasing heart failure in patients with valvular heart disease is conducive to hypoxia and damage to peripheral tissues, including kidney damage, even before a possible cardiac surgery. It may also indicate that the physiological reserves of individual organs are reduced, and particular organs are more sensitive to unfavorable conditions in the perioperative period, such as the use of extracorporeal circulation, decreases in blood count parameters and variable blood pressure values, which may favor the development of AKI in the early stages of postoperative period. Therefore, taking into account the results of the above study as well as numerous previous studies, it should be emphasized that the determination of NT-proBNP may be useful both during the observation of patients with valvular heart disease and the selection of the optimal time for surgical intervention of valvular heart disease, as well as a useful biomarker during the management of the patient for perioperative, taking into account the increased risk of events in the periprocedural period, such as postoperative hemodynamic instability, cardiogenic shock requiring extracorporeal membrane oxygenation, AKI or MODS in patients with high preoperative NT-proBNP values [19,38,39]. 

The presented study has some potential limitations. This is a single-center study with a limited number of patients included in the study and a short follow-up period. In future studies, expanding the study group and increasing the number of centers included in the study may allow confirmation of the results obtained. Further research is necessary to assess the pathomechanisms and predictors of the development of acute kidney injury in patients treated with cardiac surgery in the early postoperative period.

## 5. Conclusions

AKI in the early postoperative period in patients undergoing heart valve replacement/repair is a fairly common complication with a high risk of death. In the presented study conducted on a group of 60 patients, AKI occurred in 50 patients, of whom 38 died. To the best of our knowledge, this is the first study describing the measurement of NT-proBNP before cardiac surgery in predicting post-operative AKI requiring renal replacement therapy. Moreover, univariate logistic regression analysis indicated postoperative hemodynamic instability as one of the important factors in the development of AKI. It seems that one of the factors predisposing to the occurrence of postoperative AKI is heart failure developing in the course of the presence of a heart valve defect. The results of both the above study and the available literature may suggest the need to monitor the development of heart failure in a patient with valvular heart disease, e.g., by assessing the level of natriuretic peptide to identify the right moment for cardiac surgery, which in turn may improve treatment results and reduce the risk of death and serious postoperative complications. 

## Figures and Tables

**Table 1:** Baseline characteristics of the study population (*n* = 607).

Preoperative Characteristics of Patients	Values All Patients	Values Patients with AKI (*n* = 50)	Values Patients without AKI (*n* = 557)	*p*-Value
Age, years *	65 ± 7	71 ± 7	65 ± 7	<0.05
Male: men, *n* (%)	350 (57)	26 (52)	324 (58)	ns
Body mass index, kg/m^2^ *	27 ± 10	27.4 ± 3.5	27.8 ± 2.8	ns
LV ejection fraction, (%) *	56 ± 12	53 ± 14	58 ± 10	<0.05
EuroSCORE II, % *	3.5 ± 1.7	3.9 ± 2.1	2.4 ± 1.2	<0.05
Atrial fibrillation, *n* (%)	242 (39)	30 (60)	212 (38)	<0.05
Chronic kidney disease (GFR < 60 mL/min/1.73 m^2^), *n* (%)	186 (30)	25 (50)	161 (28)	<0.05
Coronary artery disease, *n* (%)	92 (15)	13 (26)	79 (14)	<0.05
Previous myocardial infarction, *n* (%)	46 (7)	5 (10)	41 (7)	ns
Stroke history, *n* (%)	33 (5)	5(10)	28 (5)	ns
Hypertension, *n* (%)	371 (61)	27 (54)	344 (61)	ns
Diabetes mellitus, *n* (%)	96 (15)	11 (22)	85 (15)	ns
Hemoglobin, g/dL *	13.7 ± 1.6	12.9 ± 1.3	13.8 ± 0.9	<0.05
GFR, mmol/L *	67 ± 17	56.9 ± 16.5	68 ± 13.5	ns
Hs-TnT, ng/L *	40 ± 11	31.4 ± 13.8	12 ± 6.8	ns
NT-proBNP, pg/mL	2063 ± 1751	3089 ± 2703	804 ± 800	<0.05
CRP, mg/dL	0.5 ± 0.2	0.5 ± 0.4	0.2 ± 0.2	<0.05
Platelets, 1000/uL *	186 ± 60	180 ± 49	187 ± 34	<0.05
Intraoperative characteristics of patients				
Aortic cross-clamp time, min *	104 ± 42	113 ± 26	87 ± 32	ns
Cardiopulmonary bypass time, min *	138 ± 57	172 ± 41	113 ± 44	<0.05
Postoperative characteristics of patients				
Postoperative major blending, *n* (%)	50 (8)	6 (12)	44 (8)	ns
Stay in the ICU longer than 2 days, *n* (%)	345 (56)	48 (96)	297 (53)	<0.05
Hemodiafiltration time, days *	4 ± 2			
Main procedures				
AVR, *n* (%)	306 (50)	15 (30)	191 (52)	<0.05
AVP, *n* (%)	10 (2)	2 (4)	8 (14)	ns
AVR + MVR, *n* (%)	56 (10)	13 (26)	43 (8)	<0.05
AVR + MVP, *n* (%)	11 (2)	1 (2)	10 (2)	ns
AVP + MVP, *n* (%)	3 (0.5)	0	3 (0.5)	ns
MVP, *n* (%)	109 (17.5)	10 (20)	99 (18)	ns
MVR, *n* (%)	107 (17.5)	7 (14)	100 (17)	ns
TVR, *n* (%)	5 (1)	1 (2)	4 (1)	ns
Concomitant procedure				
CABG, *n* (%)	92 (15)	13 (26)	79 (14)	<0.05

The values are represented by the mean * and a measure of the variation of the internal standard deviation. Abbreviations: AVR—aortic valve replacement, AVP—aortic valve plasty, CABG—coronary artery bypass graft, CRP—C-reactive protein, GFR—glomerular filtration rate, Hs-TnT—high sensitivity troponin t, ICU—intensive care unit; LV—left ventricle, MVR—mitral valve replacement, MVP—mitral valve plasty, NT-proBNP—n-terminal of the prohormone brain natriuretic peptide and TVR—tricuspid valve replacement.

**Table 2:** Analysis of predictive factors for the occurrence of postoperative renal replacement therapy.

	Univariate Analysis			Multivariate Analysis		
Variable	Odds Ratio	95% Cl	*p*-Value	Odds Ratio	95% Cl	*p*-Value
Age, years	1.067	1.033–1.103	<0.001	1.037	1.001–1.075	0.04
Chronic renal failure (GFR < 60 mL/min/1.73 m^2^), *n* (%)	1.488	1.004–2.207	0.04			
Hemoglobin, g/dL	0.646	0.541–0.770	<0.001			
NT-proBNP, pg/mL	2.226	1.718–2.884	<0.0001	1.406	1.015–1.949	0.04
CRP, mg/dL	1.585	1.274–1.974	<0.001	1.523	1.171–1.980	0.001
EuroSCORE II,%	1.172	1.098–1.251	<0.001	1.090	1.014–1.172	0.01
Atrial fibrillation, *n* (%)	1.488	1.042–2.207	0.04			
Hemodynamic instability, *n* (%)	13.882	6.132–31.428	<0.001			
Stay in the ICU longer than 2 days, *n* (%)	20.848	5.003–86.871	<0.001	9.077	2.026–40.663	0.004
LV ejection fraction, (%)	0.971	0.949–0.994	0.001			
Coronary artery disease, *n* (%)	2.125	1.080–4.182	0.02			

Abbreviations: CRP—C-reactive protein, GFR—glomerular filtration rate, ICU—intensive care unit, LV—left ventricle, NT-proBNP—n-terminal of the prohormone brain natriuretic peptide.

## Data Availability

Research data available from the author of the publication.
